# Adaptive binding and selection of compressed 1,ω-diammonium-alkanes *via* molecular encapsulation in water†
†Electronic supplementary information (ESI) available. CCDC 1040388–1040390. For ESI and crystallographic data in CIF or other electronic format see DOI: 10.1039/c4sc03945a



**DOI:** 10.1039/c4sc03945a

**Published:** 2015-01-12

**Authors:** Dan Dumitrescu, Yves-Marie Legrand, Eddy Petit, Arie van der Lee, Mihail Barboiu

**Affiliations:** a Adaptive Supramolecular Nanosystems Group , Institut Européen des Membranes , ENSCM/UMII/UMR-CNRS 5635 , Pl. Eugène Bataillon , CC 047 , 34095 Montpellier, Cedex 5 , France . Email: mihail-dumitru.barboiu@univ-montp2.fr

## Abstract

Guest molecules confined inside hollow molecular assemblies and thus protected from their environment can show unexpected structural behavior or special reactivity compared to their behavior in a bulk, unprotected environment. A special case is the coiling behavior of variable-length alkane chains in rigid hydrogen-bonded molecular cages. It has been found before that coiling may occur in such circumstances, but no experimental evidence concerning the exact conformation of the chains has yet been presented. We reveal in this study the self-assembly of a molecular cage in water and the crystalline state from three distinct components in which linear 1,ω-diammonium-alkanes chains are confined with different degrees of compression. The exact coiling behavior is determined from atomic resolution X-ray diffraction showing crenel-like conformations in the compressed state. Chemical selection can be obtained from mixtures of alkane chains *via* the encapsulation of kinetically stable conformations observed during the encapsulation of pure components. Moreover, it was found that uncompressed and compressed chains can be competitively trapped inside the capsule. These findings may provide insight in areas to a better understanding of biological processes, such as the fatty acid metabolism.

## Abstract 2

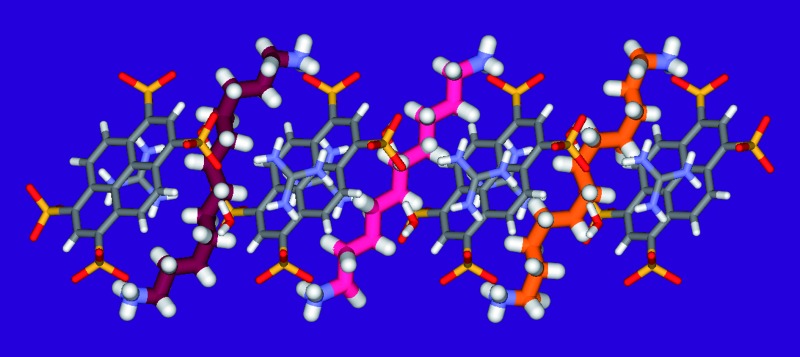
Alkane chains may be encapsulated inside rigid crystalline capsules, adopting specific conformations of different levels of compression that are sufficiently kinetically stable under the confined conditions, to allow a conventional structure determination by X-ray diffraction.

## Introduction

Encapsulation chemistry is a fascinating domain, as the properties of the guests are generally different inside host capsules compared with the bulk solution. Crossing the solution/capsule barrier, unexpected phenomena can be observed within a “compartmentalized” chemical space, opening the door for new emergent areas of chemistry and physics under confined conditions.^1–6^ Compartmentalization is also a basic feature of biological processes, as most of the physiological processes occur in cells and depend on selective exchanges of metabolites between the cell and its exterior.^7^

One of the intriguing phenomena observed to date, concerns the compression of *n*-alkanes inside self-assembled H-bonded capsules in water. Attempts to encapsulate alkane molecules in confined spaces have generally furnished very interesting details on their constitutional flexibility, inducing synergetic dynamic shape changes of the capsule/host system.^8–11^ Internal order can in some cases be reinforced by synergetic supplementary strong interactions between guests and host capsules.^11^ Stabilization of large alkanes, adapting their conformation in smallest capsules, has been demonstrated to shed light evidence of constrained conformations and give answers on how alkane compression occurs.^8^ Dynamic selection of congruent alkyl-ester hosts can be adaptively obtained on guest encapsulation.^9^

We recently reported the exact behaviors of compressed alkanes at the molecular level constrained within crystalline molecular capsules. They were determined from atomic resolution X-ray diffraction and confirmed the previous molecular modeling as well as the spectroscopic NMR studies in aqueous solution.^12^ For this reason, we designed a crystalline superstructure “Pyrene box” from available commercial materials, containing the 1,3,5,8 pyrenetetrasulfonate anions, **PTS^4–^**, the guanidinium cations, **G^+^** and 1,ω-diammonium-alkanes.

The confinement of the 1,10-diammonium-decane, **1**, 1,11-diammonium-undecane, **2** and 1,12-diammonium-dodecane, **3** within the **PTSG** host-matrix has been readily realized in aqueous solutions of **G^+^Cl^–^**, **PTS^4–^4Na^+^** and **1**, **2** or **3** resulting in the formation of the inclusion single crystals of **PTSG{1}**, **PTSG{2}** and **PTSG{3}**, respectively. These preliminary results showed crenel-like conformations of the larger alkanes in the compressed states. H-bonding and van der Waals interactions between alkane guest and the resulted capsule host are strongly and synergistically contributing for the assembly of the alkane-capsule systems in water.^12^ At his point, two questions arise: (a) how important are the presence of molecular components and how does they contribute to the stabilization of the overall host–guest superstructure? (b) How selective is alkane encapsulation within competitive conditions?

In order to answer these questions, herein we expanded our initial study to different capsule systems and/or to competitive encapsulation screenings of mixtures of alkanes. To our surprise, we observed that adaptive encapsulation occur and both highly compressed and non-compressed chains are equally favored. This is not so expected, as it would imply that a hydrophobic pocket of a given finite volume would not be specific for a particular alkane guest. A brief survey of the literature data available for biological entities indicated a similar lack of selectivity occurring in the fatty acid metabolism. More specifically, medium chain acyl-CoA dehydrogenase shows a weak specificity towards C8 alkane chains, but can also competitively encapsulate C10, C12 and C14 alkane chains under certain conditions.^13^

## Results and discussion

### Strategy

Our previous experience in the confinement of unstable species inside a crystalline guanidinium–sulfonate, **GS** molecular flask, led us to consider a similar approach for compressing alkanes.^5^ Alkanes, however, are not a preferred building blocks in crystal engineering, due to their very high number of possible conformations generating disorder and their general lack of affinity.

**Fig. 1:**
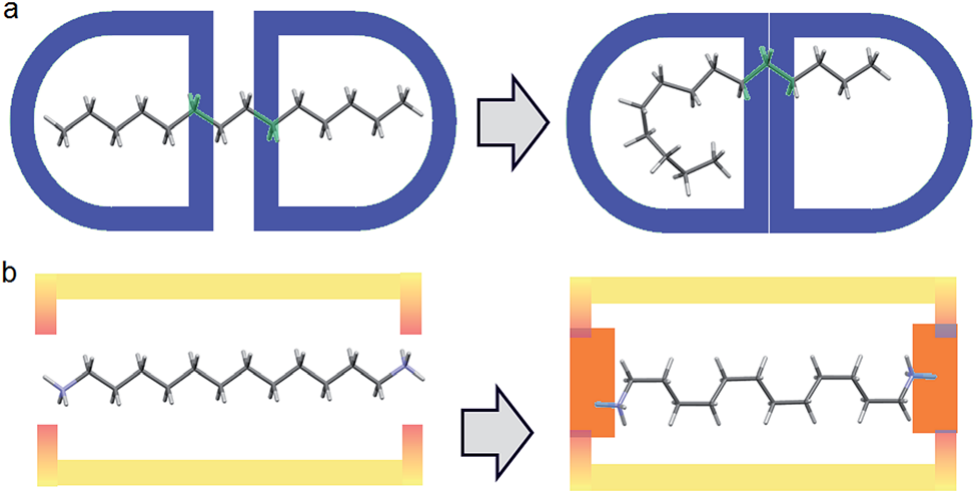
(a) Free motion of unfixed of alkane inside a capsule host; (b) Diammonium alkane encapsulation, fixed at the ends with fewer degrees of freedom *via* H-bonding and charge interactions.

In practice, it is very difficult to co-crystalize a long alkane, especially in a precise way. Furthermore, from the chemical point of view, alkanes are not very compatible with **GS** networks. In order to generate the **PTSG** {1,ω-diammonium-alkanes} host–guest, we found it important to play on the hydrophobic/hydrophilic balance. Using water as solvent, the encapsulating components had to have both hydrophobic and hydrophilic behaviors; the 1,3,5,8-pyrene-tetrasulfonate **PTS^4–^** anion being a good candidate, ideal to make H-bonded ionic complexes with donor H-bonding cationic species.^5^ Guanidinium cations, **G^+^** were chosen as suitable counter-ions and H-bond donors. The 1,ω-diammonium-alkanes have been used for their higher solubility in water compared with alkanes. Moreover the ammonium groups might be considered as tethering points by interacting with the sulfonate groups of **PTS^4–^** anions *via* H-bonding and ionic interactions (Fig. 1). A series of 1,ω-diammonium-alkanes of varying lengths have been chosen to screen the molecular chain behaviours inside a fairly robust **PTSG** cage, with fixed dimensions.

### NMR studies in aqueous solution

**Fig. 2:**
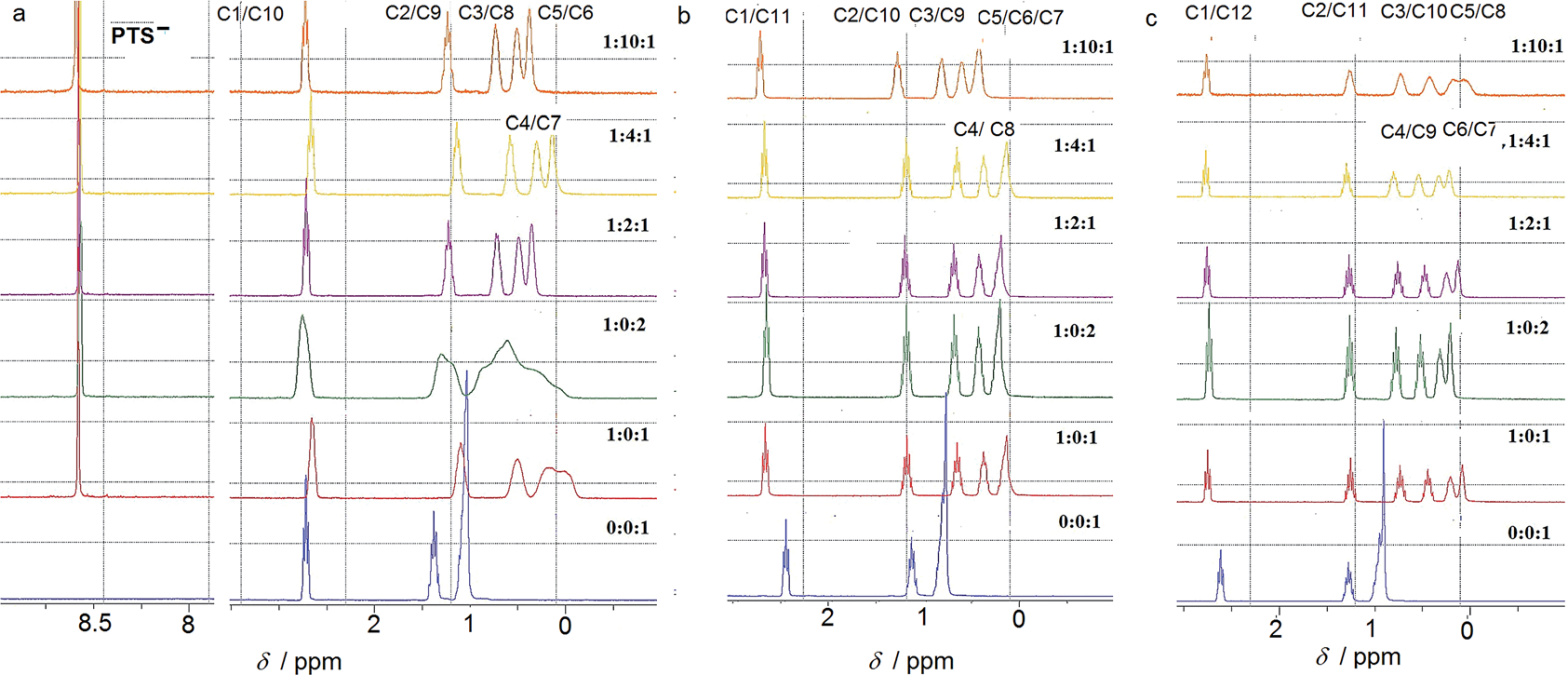
^1^H-NMR spectra at different molar ratios of **PTS^4–^** : **G^+^** : 1,ω-diammonium alkane in D_2_O at 25 °C for (a) 1,10-diammonium-decane, **1**, (b) 1,11-diammonium-undecane, **2** and (c) 1,12-diammonium-dodecane, **3** guest compounds.

The ion-pairing proxy interactions between 1,ω-diammoniumalkanes **1–3** and **PTS^4–^** anions can be detected in aqueous solution. The formation of **PTS{1–3}_2_** and **PTSG{1–3}** complexes is confirmed by the upfield shifts of the protons of encapsulated **1–3** when compared with the spectra of **1–3** recorded in the absence of **PTSG** capsule (Fig. 2). In all cases the prevalent interaction observed is the ion-pairing between **PTS^4–^** and the 1,ω-diammonium-alkanes, even in the absence of **G^+^**, which results in a shielding of the methylene groups shifts in the middle of the chain by about 1 ppm (Fig. 2). The broad upfield protons signals, showing –C–H(alkane chains)/π(PTS) interactions are reminiscent of important dynamic behaviors of alkane chains within the ion-pairing system **PTS{1}_2_** but not for **PTS{2}_2_** and **PTS{3}_2_**. This conformational motion of dication **1** interacting with **PTS^4–^** anions, is largely reduced in the presence of **G^+^** cations, as shown by the presence of sharp peaks (Fig. 2), reminiscent with the strong H-bonding/ion-pairing interactions in aqueous solution. This means that the presence of the **G^+^** cation is required for the stabilization of the “Pyrene box”, in which the motion of alkane chains is restricted. The slight upfield shift decrease with increasing length of alkane chains of **2** to **3** and can be explained only if the alkane chains adopt coiled conformations within the **PTSG** capsule, spatially allowing the folding within the confined space, as previously observed.^15^ The signal corresponding to the methylene groups in the 1 and ω terminal positions of the alkane chain remains unchanged in the presence **PTS^4–^**. (Fig. 2). This is in good agreement with the crystal structures (see below), where the middle of the chain is shielded by the pyrene rings and the terminal atoms are close to the edge, almost out of the box. In general, higher concentrations of **G^+^** resulted in a stronger shielding, with the exception of 1,10 diammonium-decane where a dynamic process is observed (Fig. 2a). The stoichiometry of the complex present in solution was determined by titration experiments. A molar ratio of 1 : 2 : 1 **PTS^4–^** : **G^+^** : **1–3** is always enough to generate the confined systems in solution. Although not clearly evident from the NMR titration experiments, it should be noted that crystals could only be obtained in **PTS^4–^** : **G^+^** : **1–3** ratios of 1 : 4 : 1, with the exception of **PTSG{1}_2_**.

**Fig. 3:**
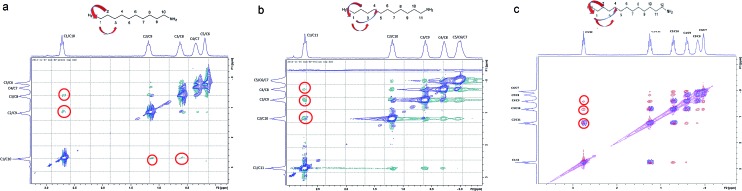
Stack between the COSY (blue) and ROESY (a and b) green, (c) (red) spectra of (a) **PTSG{1}**, (b) **PTSG{2}** and (c) **PTSG{3}**. Long range spatial interactions can be observed between C1–C2, C1–C3, C1–C4 carbon atoms away.

**Fig. 4:**
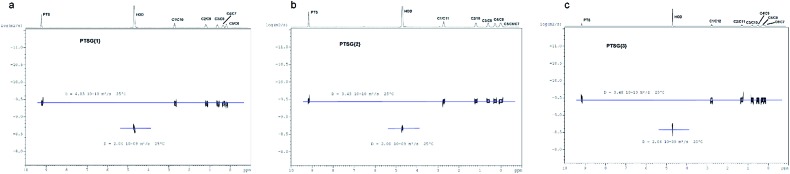
DOSY spectra of (a) **PTSG{1}**, (b) **PTSG{2}** and (c) **PTSG{3}**.

The compression of the alkane chains is confirmed by COSY/ROESY experiments (Fig. 3). In the case of **PTSG{1}**, the alkyl chain appears to have an elongated zig–zag conformation, with no correlation observed on a distance larger than 2 carbons (distinct NOE effects are found between the protons H_1_ to H_3_) (Fig. 3a). In the case of **PTSG{2}** (Fig. 3b) and **PTSG{3}** (Fig. 3c) spatial correlations are visible up to 4 carbon atoms away (distinct NOE effects are found between the protons H_1_ to H_4_). Furthermore, the DOSY spectroscopy (Fig. 4) showed that only one dimensionally constant capsule is present in solution for the all **PTSG{1–3}** systems (Table 1). The calculated diffusion coefficients get progressively smaller from **PSTG{1}** to **PTSG{3}**, which is consistent with a mass increase of the capsule (Table 1).

**Table 1:** DOSY diffusion coefficients for **PTSG{1–3}**. For weighing, a diffusion coefficient of 2.00 × 10^–9^ m^2^ s^–1^ was considered for HOD at 25 °C

	**PTSG{1}**	**PTSG{2}**	**PTSG{3}**
Complex diffusion coeff. (m^2^ s^–1^)	4.03 × 10^–10^	3.43 × 10^–10^	3.48 × 10^–10^
HOD diffusion coeff. (m^2^ s^–1^)	2.04 × 10^–9^	2.00 × 10^–9^	2.06 × 10^–9^
Weighed complex diffusion coeff. (m^2^ s^–1^)	3.95 × 10^–10^	3.43 × 10^–10^	3.38 × 10^–10^

On the basis of these NMR data one may conclude that the molecular alkane strands of **1–3** are confined within **PTSG{1–3}** cages, which are stable in water. Based on long distance interactions observed in COSY/ROESY experiments, alkane strand of **1** is stable in the elongated and slightly compressed conformations, while alkane strands of **2** and **3** adopt in aqueous solution the compressed conformations.

### X-ray crystal structures of **PTSG{1–3}_2_**

**Fig. 5:**
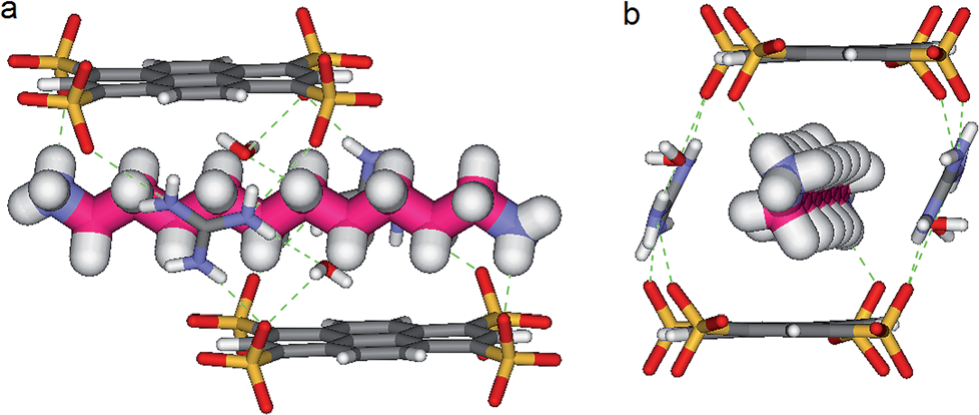
X-ray structure of **PTSG{1}**: (a) Side and (b) Front view of the **PTSG{1}** inclusion complex. 1,10-diammonium-decane guest, 1 (magenta) presents inside the **PTSG** box, an uncompressed zig–zag architecture.

**Fig. 6:**
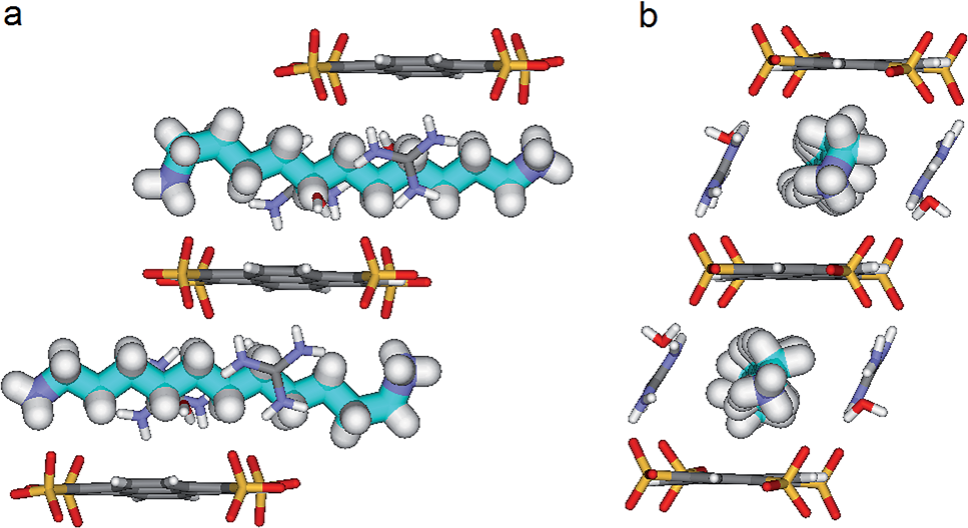
X-ray structure of **PTSG{2}**: (a) Side and (b) Front view of the **PTSG{2}** inclusion complex. 1,11-Diammonium-undecane guest **2** (cyan), presents inside the **PTSG** box, an slightly compressed architecture with a gauche conformation at the terminal part of the chain.

**Fig. 7:**
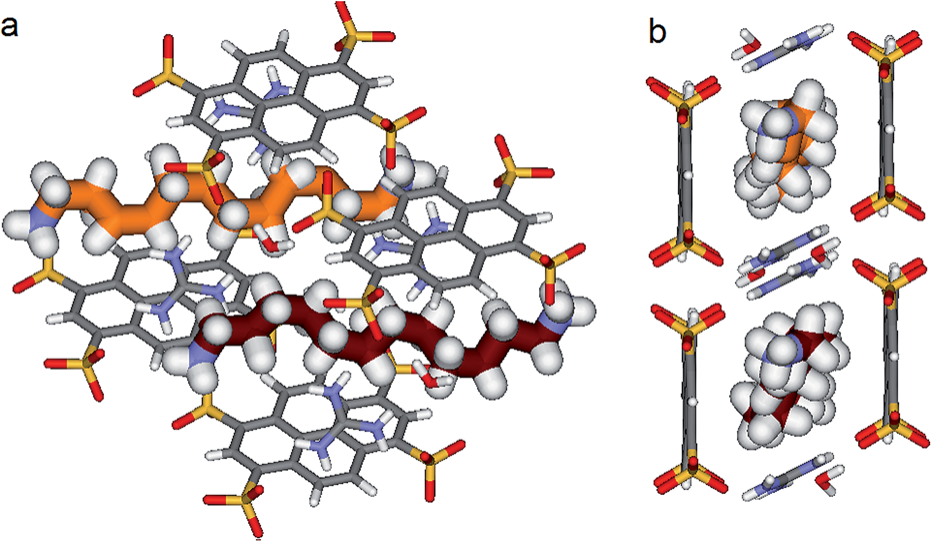
X-ray structure of **PTSG{3}**: (a) Side and (b) Front view of the **PTSG{3}** inclusion complex. 1,12-diammoniumdodecane, **3** presents inside the **PTSG** box, two highly compressed architectures **3a** and **3b** having 6 gauche (orange) and having 4 gauche (red-violet) conformations, respectively.

In the crystal, the “Pyrene box” results from the self-assembly *via* H-bonding (*d*_O···H–N_ of 2.00 Å) and charge interactions of two sulfonate moieties from **PTS^4–^** anions and of two **G^+^** cations. This network is reinforced by two bridging water molecules, which are simultaneously H-bonded to **G^+^** (*d*_O···H–N_ of 2.00 Å) and to **PTS^4–^** (d_S···H–O_ of 1.98 Å), playing a critical role in organizing the partners *via* three sulfonate moieties on the faces of the “Pyrene box”. The 1,ω-diammonium-alkanes, **1–3** are confined within inner space of parallelepipedic boxes defined between two **G^+^** and two **PTS^4^**, while each ammonium moieties of 1,ω-diammoniumalkanes form two anchoring H-bonds (*d*_N–O_ = 1.93 Å) with two other sulfonate moieties themselves non bonded to **G^+^** cations (Fig. 5–7). It results that the 1,ω-diammonium-alkanes, **1–3** practically immobilized *via* synergetic H-bonds and hydrophobic interactions with the pyrene walls, have well-defined electron density sites in the Fourier maps, with any observed motional disorder for carbon atoms. All combined H-bonding and hydrophobic host–guest interactions are vital for the encapsulation and insulation as well as for the low mobility of the alkane guest chains in the “Pyrene Box”. The average distance between the two planes of non H-bonded sulfonate groups is 13.80 Å. 1,10-Diammonium-decane, **1** fits perfectly the length of the “Pyrene box”, with each ammonium moiety bonding to a sulfonate moiety of **PTS^4–^**. The chain adopts a classical ‘zig–zag’ elongated conformation, with an average sp^3^carbon-sp^3^carbon length of 1.52 Å in the expected range (Fig. 5).^14^

By increasing the alkane chain length by only one carbon atom, the 1,11-diammonium-undecane chain becomes longer than the length of the box and adopts an asymmetrical conformation, **2** having one gauche conformation at the end of the chain (Fig. 6). X-ray structure determinations at variable temperature were performed in order to better understand the dynamic behavior of the compressed chains of **2** within the box. The undecane chain in **PTSG{2}** show dynamic behaviors at temperatures higher than 140 K and becomes disordered and symmetrical at room temperature. We interpreted this reversible flipping change as the rapid movement of the gauche conformation at room temperature from one end of the chain to the other.^12^

Another incremental increase to 1,12-diammonium-dodecane, **3** leads to an even more compressed alkane chain in **PTSG{3}** (Fig. 7). Careful crystallographic modelling allowed us to identify two co-existing conformers **3a** and **3b** of the chain in a 2 : 1 ratio. Both cases show high compression, with conformer **3a** having 6 gauche conformations and **3b** having 4 gauche, respectively.

Interestingly, no dynamic behaviors, leading to the reversible change between conformations, were observed in the case of both **PTSG{1}** and **PTSG{3}**, even at temperatures as high as 340 K. This is consistent with the “natural” stability of the extended decane chain of **1**, perfectly fitting inside the “Pyrene box”, but totally unexpected for the two similarly disordered dodecane chains **3a** and **3b**, probably too constrained to move.

The compression under confinement results in a decrease of the overall length of the natural uncompressed ‘zig–zag’-type 1,12-diammonium-dodecane, **3** and 1,11-diammonium-undecane **2** chains: from 16.534 Å to 14.465 Å (**3a** and **b**) and from 15.036 Å to 14.239 Å (**2a** and **b**), respectively.

Non-Covalent Interactions (NCI) plots^15^ showed that the confined alkane chains present strong van der Waals contacts with all the inner surfaces of the “Pyrene box” and the compressed states present attractive hydrogen–hydrogen contacts inside the gauche conformations. A closer examination of these ‘crenel’ conformations **3a** and **3b** revealed a distance of only 2.24 Å between the hydrogens inside (*i.e.* the hydrogens in positions 1 and 4, 3 and 6 *etc.*), shorter than the sum of their van der Waals radius (approx. 2.5 Å)-see ref. 12 for details.^16^

With all this in mind, it remains to determine how strong are the stabilizing interactions and how much does they contribute to the overall structure and crystallization process? The combination of H-bonding and hydrophobic interactions is not immediately apparent, as one cannot estimate quantitatively the synergetic interaction strengths.

**Fig. 8:**
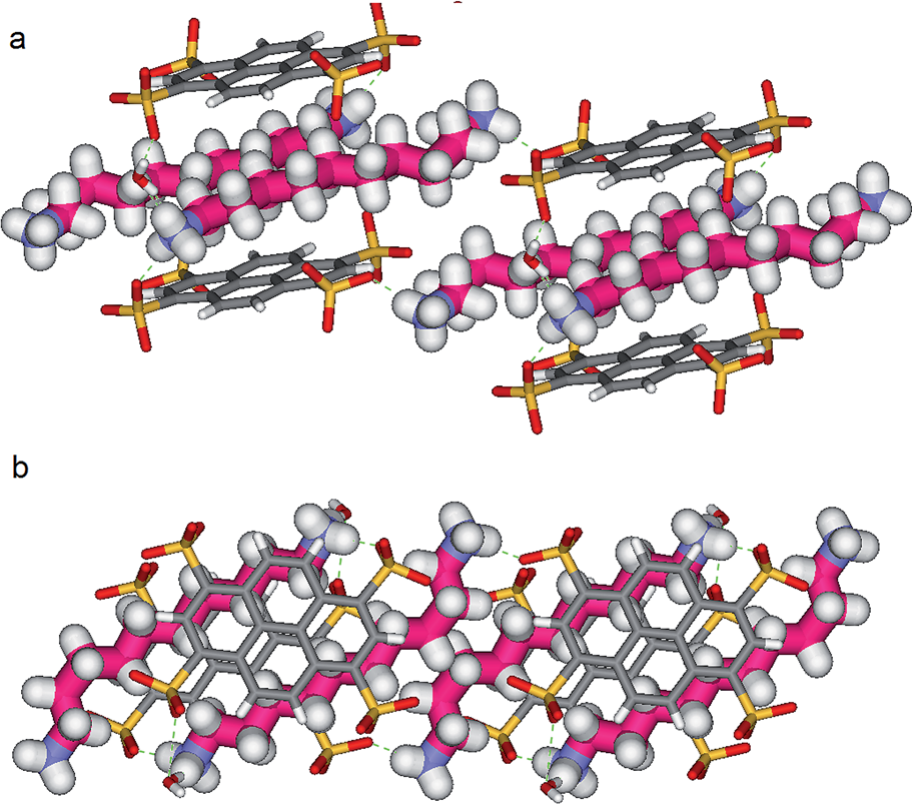
X-ray structure of **PTS{1}_2_**: (a) side and (b) front view of the **PTS(1)_2_(H_2_O)_4_** inclusion complex. 1,11-diammonium-dodecane guest **2** (magenta) presents inside the “pseudoPyrene box” a slightly compressed architecture with the gauche conformation present between the C2–C3 of the chain.

We know that the Pyrene box is always forming in the presence of **PTS^4–^**, **G^+^** and 1 eq. of 1,ω-diammonium-alkanes **1–3**. Exchanging the **G^+^** with diammonium cations **1–3** by increasing their ratio to 2 or 4 eq., only **PTS{1}_2_** single crystals are obtained, while the corresponding to **PTS{2}_2_** and **PTS{3}_2_** are precipitating. The absence of the guanidinium cations induce a new “face to face” relative arrangement of the **PTS^4–^** platforms into the novel crystalline-“pseudoPyrene box” so that the average distance between the two planes of the sulfonate groups is drastically reduced to 7.11 Å. The 1,10-diammonium-decane, **1** much longer to fit the new constraints is H-bonded to two **PTS^4–^** platforms from neighboring “pseudo-Pyrene boxes” resulting in the formation of polymeric superstructures of H-bonded alkane chains bridging the “pseudoPyrene boxes” (Fig. 8). The “pseudoPyrene box” is reinforced by two bridging water molecules, which are simultaneously H-bonded to ammonium cations (*d*_O···H–N_ of 1.97 Å) and to **PTS^4–^** (*d*_O···H–N_ of 1.98 Å), playing a critical role in organizing the **PTS^4–^** anions in the face-to-face configuration of the “pseudoPyrene box”. This is inducing a small compression behavior of the decane chain adopting an asymmetrical conformation, having one gauche conformation between C2–C3 atoms of the chain (Fig. 8).

### Competitive crystallization experiments

Finally, we decided to perform competitive crystallization experiments in the presence of **PTS^4–^** : **G^+^** (1/4, mol/mol) and equimolar mixtures **1** + **2**, **2** + **3** and **1** + **3** and (**1** + **2** + **3**), respectively (Table 2). They are excellent candidates for the generation of dynamic supramolecular libraries in solution, resulting in the formation of interchanging equilibrating mixture of components of Pyrene or pseudoPyrene boxes of different compositions, which might converts into unique/specific solid state species *via* a kinetically irreversible crystallization process.^17,18^

**Table 2:** Competitive crystallization experiments in the presence of **PTS4^–^** : **G^+^** (1 : 4, mol/mol) components

Mixture of 1,ω-diammoniumalkanes	Resulted crystallized structure
**1** + **2**	**PTS{1}_2_**
**2** + **3**	Precipitate
**1** + **3**	**PTSG{1}_0.62_{3}_0.38_**
**1** + **2** + **3**	**PTSG{1}_0.62_{3}_0.38_**

Crystals of PTS{1}_2_*, a polymorph of the original **PTS{1}_2_** suitable for X-ray analysis were formed from a mixture of **PTS^4–^** + **1** + **2**, while the mixture **PTS^4–^** + **2** + **3** forms a precipitate within minutes. Interestingly, in the PTS{1}_2_* structure the 1,10-diammonium-decane, **1** present the same conformation with a gauche conformation between the C2–C3 of the chain (Fig. 8), but the packing behaviors are different (Fig. S1†). The selective crystallization of **PTS{1}_2_** against **PTSG{1}** can be explained by lower solubility of – “pseudoPyrene boxes” in **PTS{1}_2_**, kinetically selected in competition with the more soluble monomeric **PTSG{1}** containing the alkane chains included in the “Pyrene box” capsule.

**Fig. 9:**
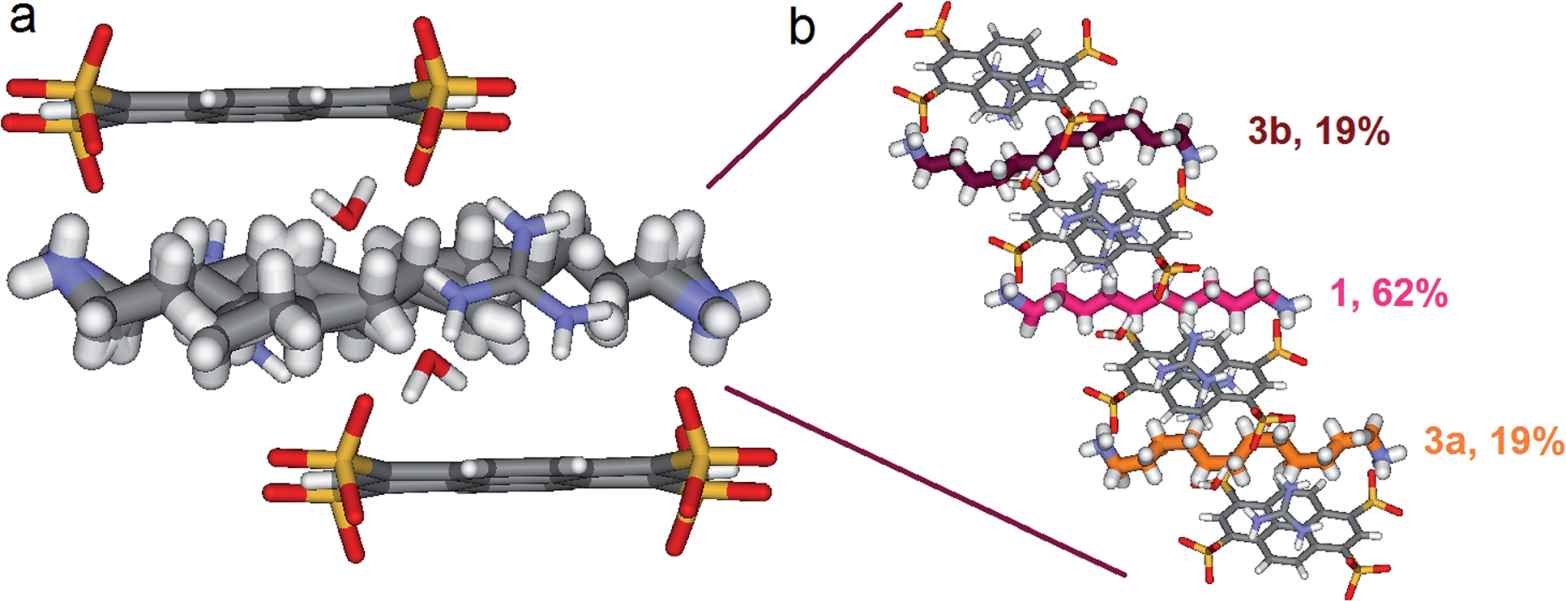
X-ray structure of **PTSG(1)_0.62_(3)_0.38_** resulted as unique crystallizing component from the reaction mixtures of **PTS^4–^** : **G^+^** (1 : 4, mol/mol) and **1** + **3** or **1** + **2** + **3**. 1,10-Diammonium, **1** (62%) and 1,12-diammonium, **3** (2 × 19%) guests are present inside the PTSG box as an elongated zig–zag **1** architecture and the two highly compressed architectures **3a** and **3b**, respectively.

Things became interesting when a hybrid **PTSG{1}_0.62_{3}_0.38_** is obtained as a unique crystallizing component from the mixtures of **PTS^4–^** : **G^+^** (1 : 4, mol mol^–1^) and **1** + **3** or **1** + **2** + **3**. The “Pyrene box” encapsulate simultaneously both non-compressed **1** (62%) and highly compressed **3a** (19%) and **3b** (19%) at the same time and in the same crystal (Fig. 9). This is based on the relative similar constitutional stabilities of un-compressed natural form of **1** and of the highly compressed form of **3**, strongly interacting the “Pyyrene box” host *via* coupled H-bonding/hydrophobic interactional algorithms, as well as several stabilizing dihydrogen contacts. This confirms the previously observed high stability of **PTSG{3}** over a large temperature domains. The N_1_–N_ω_ distance is 14.031 Å, a mean of the distance in the case of **PTSG{1}** (13.803 Å) and the case of **PTSG{3}** (14.467 Å). Dimensional anchoring and host–guest complex interaction, coupled with hydrogen–hydrogen interactions, are robust enough to induce simultaneous encapsulation of **1** and **3**.

## Conclusion

Molecular encapsulation of the alkane chains has long intrigued chemists on account of their strained geometries and variable adaptive behaviors to form dynamic molecular capsules, but these compounds or their derivatives have most often eluded crystallographic characterization.

In this paper we highlighted that the alkane chains may be successfully constrained/anchored inside a rigid crystalline capsules. They adopt specific conformations at different levels of compression depending on their dimensional behaviors that are sufficiently kinetic stable under the confined conditions, to allow a conventional structure determination by X-ray diffraction. These results not only shows that chemists are able to tune, from inside, physical properties (pressure) at the molecular scale, but also provides new hints for the, so far, not fully understood conformations of long alkane chains under confined conditions. The undecane chain in **PTSG{2}** shows an interesting movement of the *gauche* conformation from one end of the chain to the other, while the decane chain of **PTSG{1}** and the dodecane chain of **PTSG{3}**, are stable in an elongated zig–zag and highly compressed crenel-type conformations, respectively.

Variable relative spatial disposition of the **PTS^4–^** platforms within the crystal induce a different dimensional disposition of the sulfonate anchoring points used for the 1,ω-diammonium-alkanes binding. The presence of **G^+^** cations induce a slightly slipped relative position of the **PTS^4–^** platforms in the “Pyrene box”, compared with their “face to face” relative arrangement in the “pseudoPyrene box”. These different capsules can select between multiple configurations of alkane chains showing different compression behaviors and a specific adaptation, through different specific confinement mechanisms. The uncompressed and compressed conformations of the 1,10-diammonium-decane, **1** under different confinement conditions in the two crystalline boxes, shed light on the constitutional adaptivity of alkane chain to environmental factors, in order to maximize the multivalent interacting/stabilizing contacts with the host capsule. Moreover, a chemical selection can be obtained from mixtures of alkane chains *via* the encapsulation of kinetically stable conformations observed during the encapsulation of pure components.

The synergetic selection of 1,10-diammonium-decane **1** and 1,12-diammonium-dodecane **3** in the same crystal structure can lead to several interesting discussions. First, under confined conditions hydrogen–hydrogen interactions might be considered not as weak repulsive as previously envisaged. Further calculations are underway to quantify this speculation. Secondly this work may form a basis for future work explaining the relatively weak selectivity of natural hosts, such as enzymes.

Furthermore, this contribution adds several new behaviors to the systematic rationalization and prediction of hydrophobic clustering in the capsules; it unlocks the door to the new compounds world paralleling that of biology.

## Experimental

All the compounds were purchased from Sigma-Aldrich and were used without further purification. Suitable crystals for all the structures were readily obtained in large quantities by slow evaporation of aqueous solutions obtained by dissolving: 50 mg 1,3,5,8-pyrenetetrasulfonate tetrasodium salt, 100 mg guanidinium hydrochloride, 30 mg alkane diamine and 50 μL HCl 37% in 1 mL H_2_O. After 1 week the crystals were filtered and dried under mild vacuum for 2 h.

### NMR experiments

^1^H NMR experiments were performed on an AVANCE 300 MHz Bruker spectrometer in D_2_O with the use of the residual solvent peak as reference. NMR titration experiments were performed by adding successive aliquots of 0.1 M D_2_O solutions of 1,ω-diammonium alkane dichloride and guanidinium to 0.4 ml 0.01 M solution of sodium 1,3,5,8-pyrene tetrasulfonate. In the case of the 2D-NMR experiments 10 mg of the corresponding crystals obtained by the method described above were dissolved in 0.5 mL D_2_O.

2D-ROESY (Rotating-frame Overhauser Effect Spectroscopy) NMR experiments were performed at 298 K applying the 180–90° selective-spinlock—FID pulse sequence. The mixing time was recorded with 300 ms. A typical proton-proton COSY45 experiment was performed (cosygpqf). Spectra were acquired with 2 Ko data points in F2 Frequency axis and 128 experiments.

### 2D-DOSY (Diffusion-Ordered SpectroscopY) NMR

2D-DOSY (Diffusion-Ordered SpectroscopY) NMR experiments were performed at 298 K with a Bruker Dual *z*-gradient probe head capable of producing gradients in the *z* direction with strength 55 G cm^–1^. The DOSY spectra were acquired with the ledbpgp2s pulse program (2D sequence for diffusion measurement using echo and led with bipolar gradient pulse).^19^ All spectra were recorded with 8 Ko time domain data points in the F2 Frequency axis and 32 experiments (F1). The gradient strength was logarithmically incremented in 32 steps from 2% up to 95% of the maximum gradient strength. All measurements were performed with a diffusion delay D (D_2_O) of 80 ms in order to keep the relaxation contribution to the signal attenuation constant for all samples. The gradient pulse length *d* (2*P30) was 5 ms in order to ensure full signal attenuation. The diffusion dimension of the 2D DOSY spectra was processed by means of the Bruker Topspin software (version 2.1).

### X-ray diffraction

All the structures have been measured on an Agilent Technologies Gemini-S four circle diffractometer using Mo-Kα radiation (*λ* = 0.71073 Å) and equipped with a Sapphire3 detector at 175 K at the joint X-ray scattering facility of the Pôle Balard at the University of Montpellier, France. The structures have been solved using the *ab initio* charge flipping method as implemented in *SUPERFLIP*.^20^ Hydrogen atom positions were determined using Fourier differential maps in the case of **PTSG{1}** and the structure of **PTSG{2a}**. Hydrogens in the case of **PTSG{2b}** and **PTSG{3}** were added geometrically. It should be noted that in all cases the ammonium hydrogens were found using Fourrier maps. All structures were initially refined using non-linear least-squares methods as implemented in *CRYSTALS*,^21^ in which the hydrogen atoms were treated as riding on their parent atoms and with *U*_iso_(*H*) constrained to in general 1.2–1.5 times *U*_eq_(*H*) that of the parent atom. The final difference Fourier maps showed in many cases residual peaks due to bonding effects (in the pyrene planes and on the sulfonate moieties), because of the in general very good resolution of the experimental data (mostly around 0.7 Å or better, where the experimental resolution is defined according to Dauter).^22^

Disorder models and temperature dependence: in the case of 1,10-diammonium decane and 1,12-diammonium dodecane the asymmetric unit contains 1/2 of a pyrene and 1/2 of a chain. This is due to a local inversion center at the middle of the alkanedyil chain. At 175 K, in the case of 1,11-diammonium undecane, this inversion center disappears and the unit cell is doubled over the *c* axis. However, at room temperature (298 K), the unit cell is again halved (*i.e.* the same as for 1 and 3). The structure of 2 below room temperature can thus be considered as a commensurate superstructure of the room temperature structure with wave vector *q* = 0.5c*RT where c*RT is the room-temperature reciprocal *c*-vector. Reflections *hkl*: *l* = 2*n*+1 are in the low-temperature phase in general much weaker than reflections *hkl*: *l* = 2*n*. The superstructure is only due to the disorder in the alkandyl chains, the **PTS^4–^** and **G^+^** moieties remaining exactly in place in neighboring unit cells. The disappearance of the low-temperature superstructure with rising temperature was monitored by plotting the ratio of the mean *I*/*σ*(*I*)_*l*=odd_ over *I*/*σ*(*I*)_*l*=even_. Structurally this means that the low-temperature superstructure has a pseudo inversion center at the middle of the undecane chain, which becomes a real inversion center, *i.e.* belonging to the space group symmetry, only at room temperature where the rapid movement of the gauche conformation from one end of the chain to the other makes that the two confirmations are even likely. This process was found to be completely reversible. The room temperature structure of **PTSG{2}** can be alternatively refined in the doubled unit cell as well as in the small unit cell (the same as for **PTSG{1}**, since the disorder is nearly complete, but because of the very faint intensity of the commensurate super reflections, the refinement is not very stable in the doubled unit cell. In the small unit cell the site occupancy factors of the two disordered parts is necessarily 0.5, because of the presence of the space group inversion center at the middle of the odd-numbered chain. The site occupancy factors of the two disordered conformers of 3 were also refined as a function of temperature, but no significant temperature dependence was observed, showing that the movement of 3 is most possibly impossible due to a too high confinement in the Pyrene box.
