# Supplementary material for: Adaptive binding and selection of compressed 1,ω-diammonium-alkanes via molecular encapsulation in water
Source: Chem Sci. 2015 Jan 12;6(3):2079–86. doi: 10.1039/c4sc03945a (PMC5654369; doi:10.1039/c4sc03945a)
Supplement: Supplementary file 1 [file SC-006-C4SC03945A-s001.pdf]

Electronic Supplementary Material (ESI) for Chemical Science.
This journal is © The Royal Society of Chemistry 2015

SUPPLEMENTARY INFORMATION FOR

Adaptive binding and selection of compressed 1,ω-diammonium-alkanes *via* molecular encapsulation in water

Dan Dumitrescu, Yves-Marie Legrand, Eddy Petit, Arie van der Lee, Mihail Barboiu*

Institut Européen des Membranes – ENSCM-UMII-CNRS 5635, Place Eugène Bataillon, CC 047, F-34095 Montpellier, Cedex 5, France. E-mail: mihail-dumitru.barboiu@univ-montp2.fr

Table S1. Crystallographic information on data collection and structure refinement

	PTS{1}$_2$	PTSG{1}	PTSG{2a}	PTSG{2b}	PTSG{3}
Formula	$C_{16}H_6O_{12}S_4$, 2($C_{10}H_{26}N_2$), 4(H_2O)	$C_{16}H_6O_{12}S_4$, $C_{10}H_{26}N_2$, 2(CH_6N_3),2(H_2O)	$C_{16}H_6O_{12}S_4$, $C_{11}H_{28}N_2$, 2(CH_6N_3),2(H_2O)	$C_{16}H_6O_{12}S_4$, $C_{11}H_{28}N_2$, 2(CH_6N_3),2(H_2O)	$C_{16}H_6O_{12}S_4$, $C_{12}H_{30}N_2$, 2(CH_6N_3),2(H_2O)
Crystal Class	Triclinic	Triclinic	Triclinic	Triclinic	Triclinic
Space Group	*P*-1	*P*-1	*P*-1	*P*-1	*P*-1
a (Å)	7.8956(4)	9.7336(8)	9.8126(4)	9.8826(4)	9.7985(3)
b (Å)	9.6557(3)	9.7556(8)	10.2605(4)	10.2939(4)	9.8928(3)
c (Å)	14.9160(6)	10.1940(7)	19.4731(7)	9.8156(4)	10.2589(4)
α (°)	77.311(3)	95.207(6)	96.737(3)	96.289(3)	94.489(3)
β (°)	81.835(4)	95.926(6)	95.763(3)	95.622(3)	97.927(3)
γ (°)	86.214(3)	95.224(7)	94.085(3)	94.839(3)	95.090(3)
Volume (Å^3)	1097.43(8)	953.91(13)	1930.36(13)	983.19(7)	976.92(6)
Z	1	1	2	1	1
Radiation type	Mo-*K*α	Mo-*K*α	Mo-*K*α	Mo-*K*α	Mo-*K*α
Wavelength (Å)	0.71073	0.71073	0.71073	0.71073	0.71073
ρ (gcm^{-3})	1.421	1.478	1.485	1.458	1.490
Temperature (K)	175	175	125	293	175
Diffractometer type	Gemini	Gemini	Gemini	Gemini	Gemini
Scan type	ω	ω	ω	ω	ω
Reflections measured	15468	36619	15112	31497	35243
Independent reflec.	4495	5743	6676	5199	5973
R_{int}	0.032	0.038	0.022	0.038	0.038
Average size (mm)	0.25x0.35x0.50	0.30x0.40x0.60	0.30x0.50x0.70	0.40x0.45x0.50	0.30x0.50x0.70
Refinement on	*F*	*F*	*F*	*F*	*F*
R-factor	0.0437	0.0353	0.0418	0.0479	0.0404
wR	0.0483	0.0299	0.0489	0.0539	0.0494
$<\sigma(I)/I>$	0.0422	0.0408	0.0368	0.0473	0.0433
Number of parameters	289	244	496	298	299
Goodness of fit	1.0944	0.9998	0.9891	1.02233	1.0533

	**PTS{1}(H_2O)$_4$**	**PTS{1}(H_2O)$_4$***	**PTSG{1}$_{0.5}${3}$_{0.5}$**
Formula	$C_{10}H_{26}N_2$, 0.5($C_{16}H_6O_{12}S_4$),2(H_2O)	$C_{16}H_6O_{12}S_4$, 2($C_{10}H_{26}N_2$), 4(H_2O)	$C_{16}H_6O_{12}S_4$, 2(H_2O), 2(CH_6 N_3), 0.6224($C_{10}H_{26}N_2$), 0.1888($C_{12}H_{30}N_2$), 0.1888($C_{12}H_{30}$ N_2)
Crystal Class	Triclinic	Monoclinic	Triclinic
Space Group	*P*-1	*I*2/c	*P*-1
a (Å)	7.8956(4)	19.4827(5)	9.7673(3)
b (Å)	9.6557(3)	7.7013(2)	9.8143(3)
c (Å)	14.9160(6)	30.1416(7)	10.2046(4)
α (°)	77.311(3)	90	94.880(3)
β (°)	81.835(4)	101.758(2)	96.822(3)
γ (°)	86.214(3)	90	95.184(3)
Volume (Å^3)	1097.43(4)	4427.63(11)	962.75(6)
Z	2	4	1
Radiation type	Mo-*K*α	Mo-*K*α	Mo-*K*α
Wavelength (Å)	0.71073	0.71073	0.71073
ρ (gcm^{-3})	1.421	1.409	1.483
Temperature (K)	175	175	125
Diffractometer type	Gemini	Gemini	Gemini
Scan type	ω	ω	ω
Reflections measured	15468	12680	14777
Independent reflec.	4468	4256	5557
R_{int}	0.032	0.029	0.027
Average size (mm)	0.12x0.18x0.36	0.30x0.40x0.50	0.20x0.50x0.55
Refinement on	*F*	*F*	*F*
R-factor	0.0433	0.0746	0.0586
wR	0.0461	0.0767	0.0649
$<\sigma(I)/I>$	0.0422	0.0438	0.0400
Number of parameters	311	301	241
Goodness of fit	1.0815	1.0486	0.9213

Table S2. Dimensions of the pyrene box in all the three cases. Length was defined as the distance between the 1 and ω- nitrogen atoms, width as the distance between the opposing guanidinium planes and height as the distance between the opposing pyrene rings. The theoretical elongated length was calculated using the Gaussian09 computation packadge at the B3-LYP level of theory.

Pyrene box	1,10-diammonium decane (1)	1,11-diammonium undecane (2)	1,12-diammonium dodecane (3)
Length (Å)	13.807	14.239	14.465
Theoretical N-N elongated length	13.810	15.036	16.534
Compression rate (%)	0	5	13
Width (Å)	7.386	7.952	7.949
Height (Å)	8.137	8.161	8.508

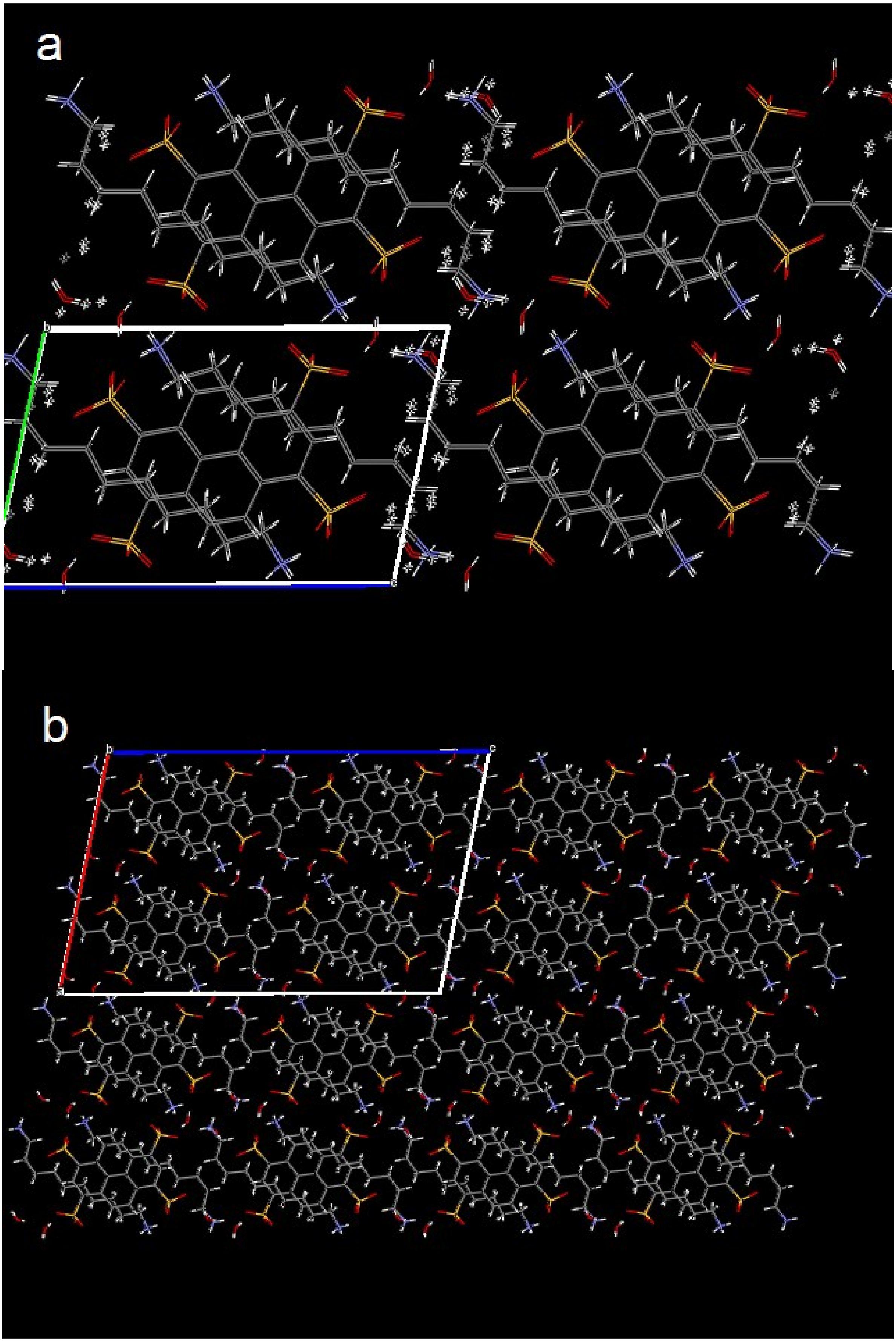

Figure S1: Comparison between the packing behaviors of polymorphs a) **PTS{1}(H_2O)$_4$** and b) **PTS{1}(H_2O)$_4^*$**

PTSG{1}

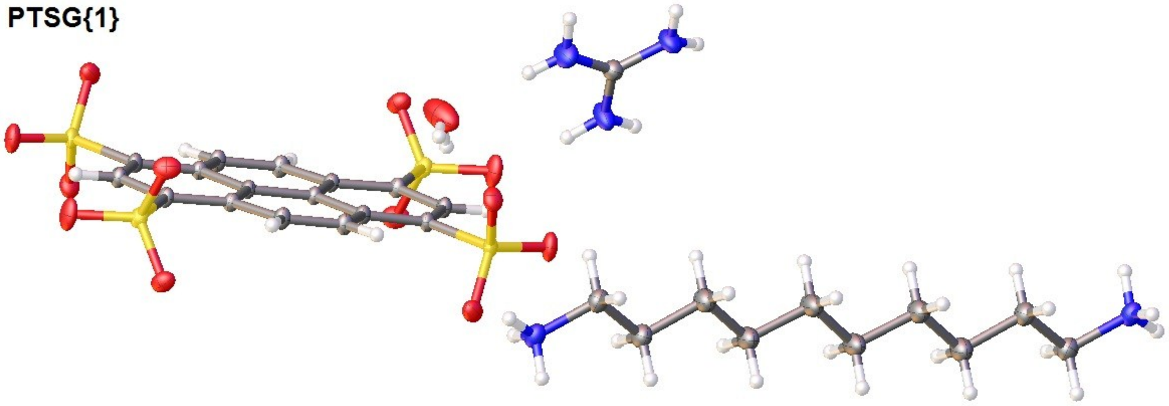

PTSG{2a}

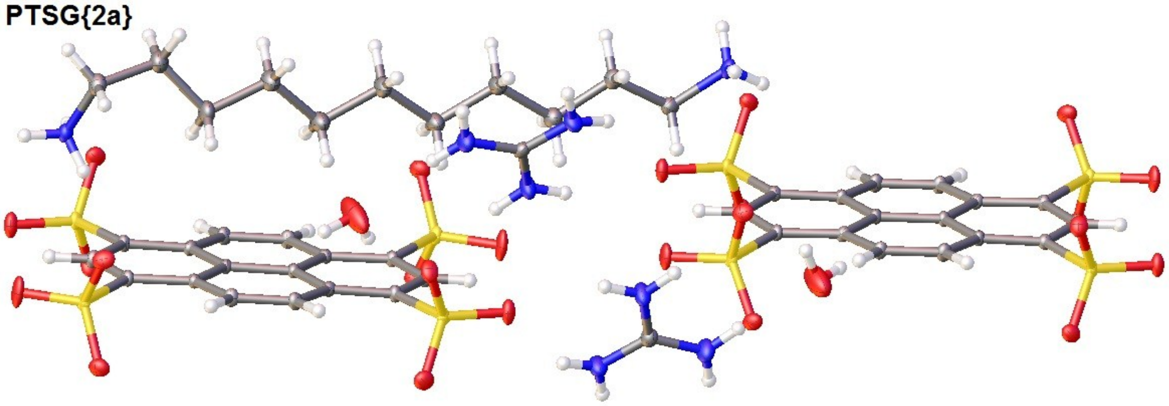

PTSG{2b}

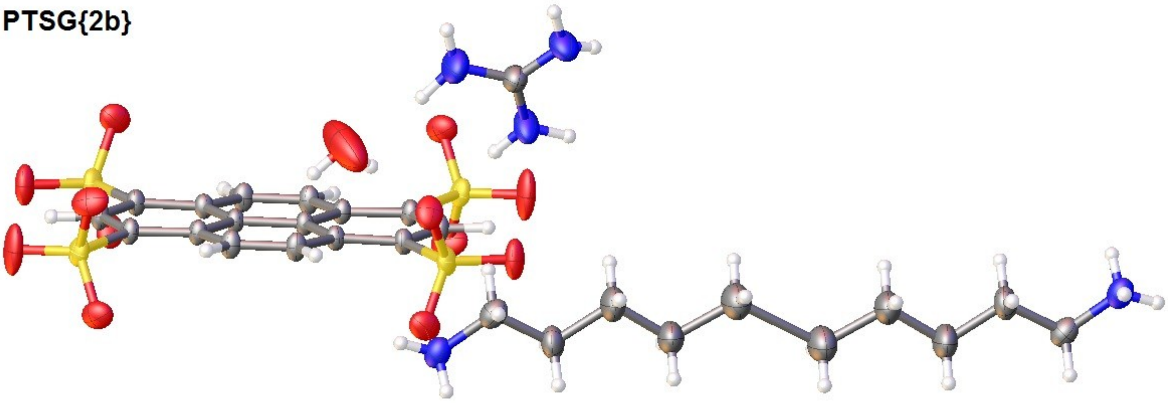

PTSG{3}

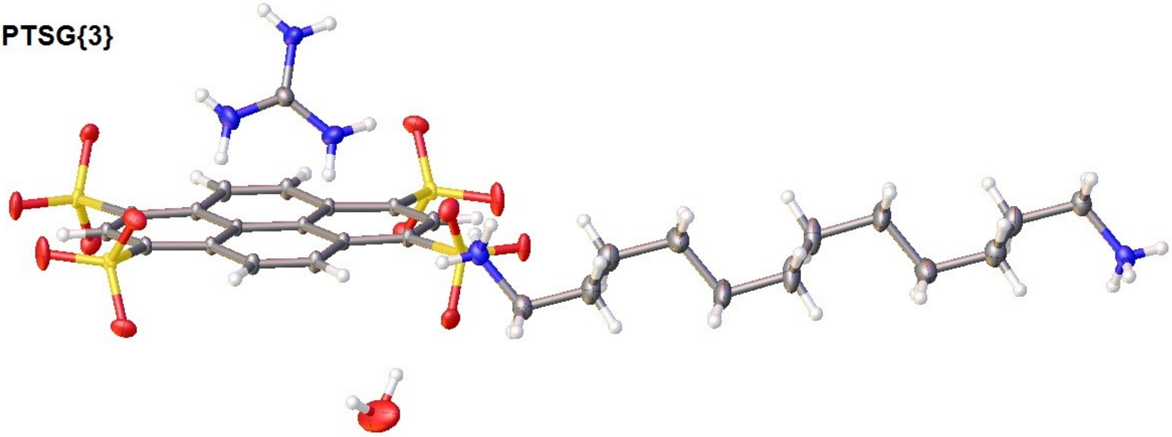

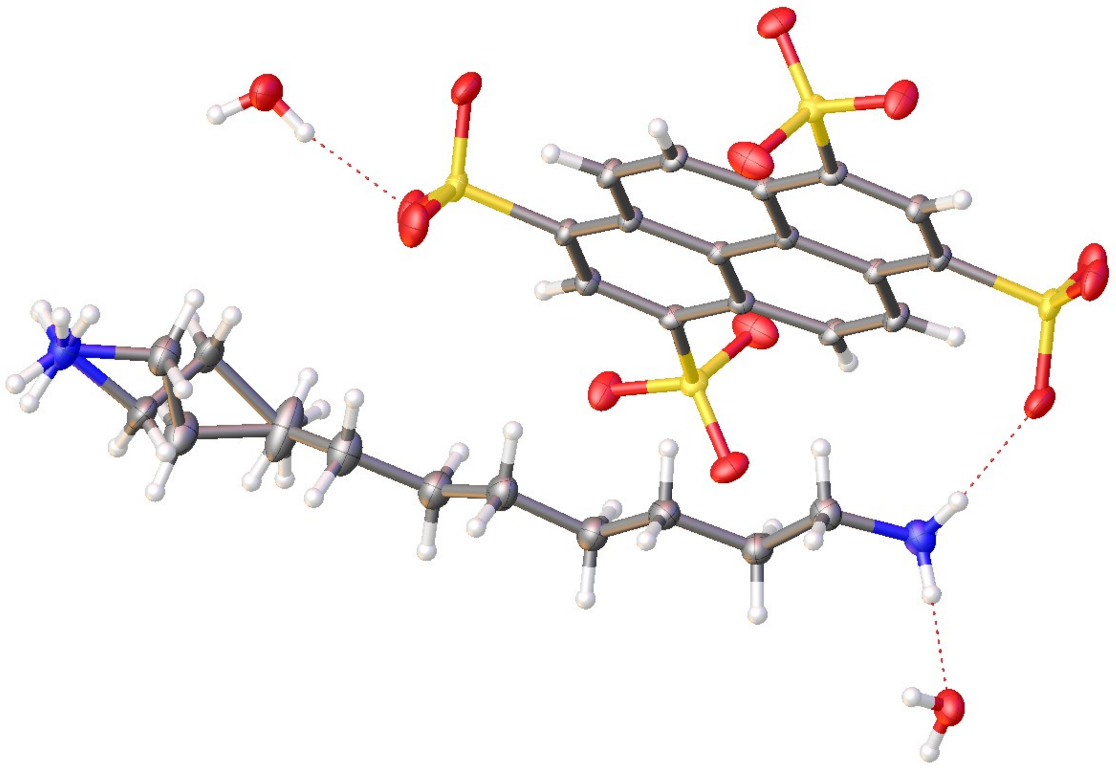

PTS{1}$(H_2O)_4$,

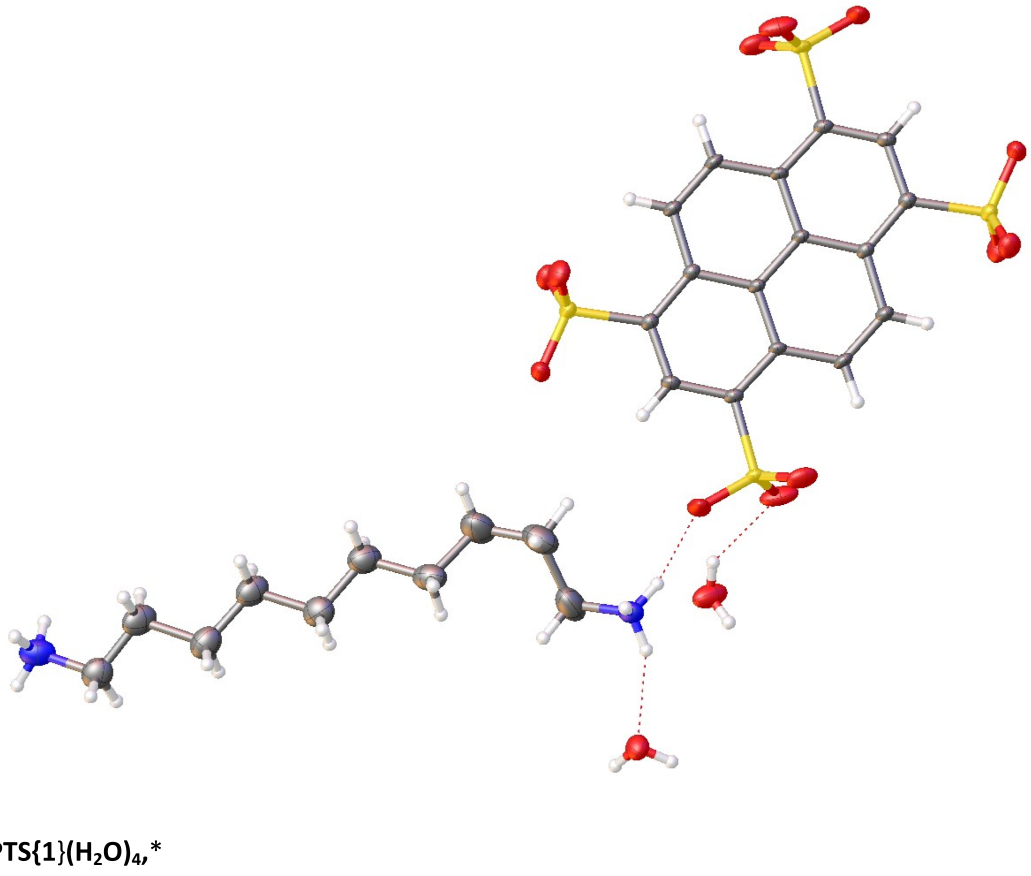

PTS{1}$(H_2O)_4$,*

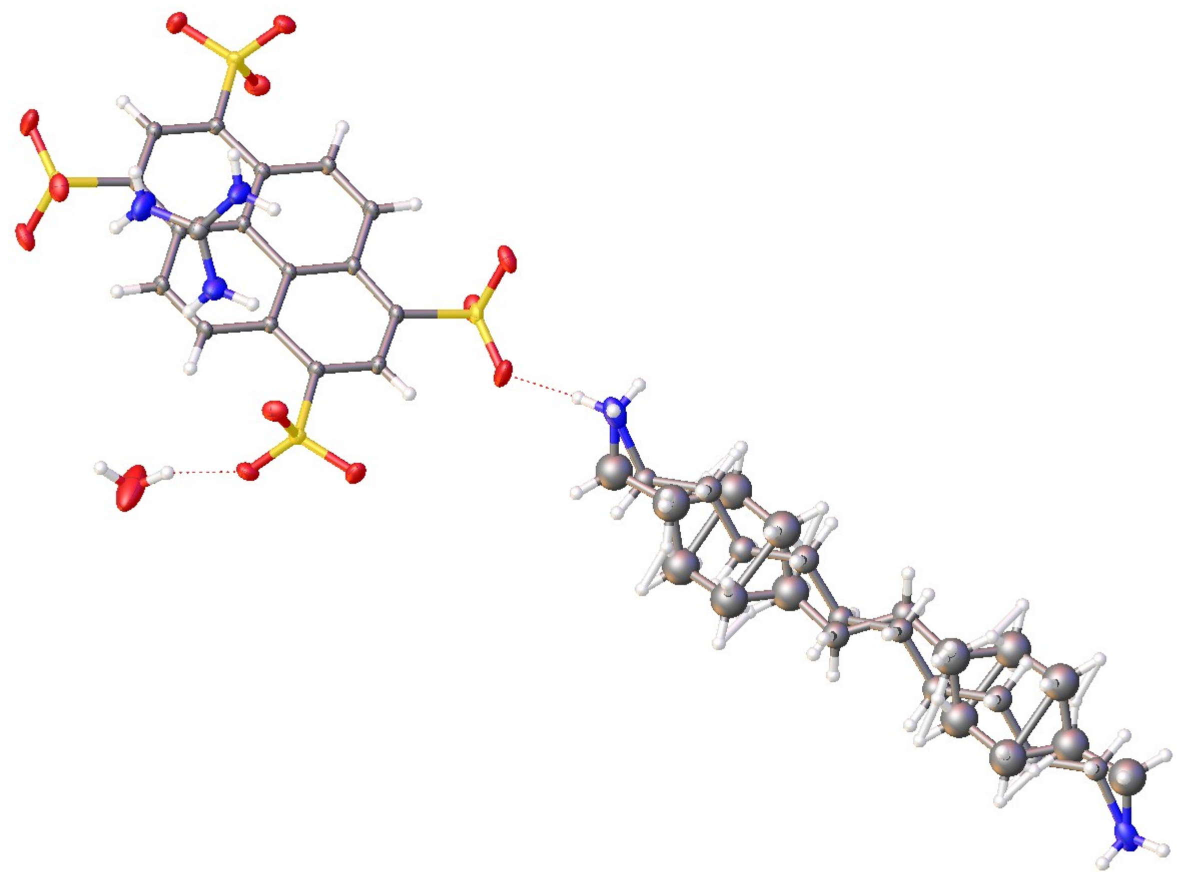

PTSG(1)$_{0.62}$(3)$_{0.38}$

Figure S2. ORTEP diagrams of structures. Thermal elipsoids are drawn at 50% probability.
